# Psychological Treatment of Depression in Primary Care: Recent Developments

**DOI:** 10.1007/s11920-019-1117-x

**Published:** 2019-11-23

**Authors:** Pim Cuijpers, Soledad Quero, Christopher Dowrick, Bruce Arroll

**Affiliations:** 1 Department of Clinical, Neuro and Developmental Psychology, Amsterdam Public Health research institute, Vrije Universiteit Amsterdam, Van der Boechorststraat 7-9, 1081 BT Amsterdam, The Netherlands; 2 Department of Basic, Clinical Psychology and Psychobiology, Universitat Jaume I, Castellón, Spain; 3 Department of Health Services Research, University of Liverpool, Liverpool, UK; 4 Department of General Practice and Primary Health Care, University of Auckland, Auckland, New Zealand

**Keywords:** Depression, Primary care, Major depressive disorder, Psychotherapy, Cognitive behavior therapy, Interpersonal psychotherapy

## Abstract

**Purpose of Review:**

We give an overview of recent developments on psychological treatments of depression in primary care.

**Recent Findings:**

In recent years, it has become clear that psychotherapies can effectively be delivered through e-health applications. Furthermore, several studies in low and middle income countries have shown that lay health counselors can effectively deliver psychological therapies. Behavioral activation, a relatively simple form of therapy, has been found to be as effective as cognitive behavior therapy. Treatment of subthreshold depression has been found to not only reduce depressive symptoms but also prevent the onset of major depression. In addition, therapies are effective in older adults, patients with general medical disorders and in perinatal depression.

**Summary:**

Psychological therapies are effective in the treatment of depression in primary care, have longer lasting effects than drugs, are preferred by the majority of patients, and can be applied flexibly with different formats and across different target groups.

## Introduction

Depressive disorders are highly prevalent, disabling, and costly disorders that are linked with considerably diminished role functioning and quality of life, medical comorbidity, and mortality [1–4]. In the past decades, considerable progress has been made in the research and development of treatments of depression in several settings, including primary care. Many different types of antidepressant medication and psychotherapy are now available that have shown to be effective in multiple randomized trials [5••, 6]. Based on these significant and positive effects, many of these treatments are included in treatment guidelines and are widely used across clinical practice.

The majority of depressed patients are treated in primary care and only a small proportion of these are referred to mental health services [7, 8]. Despite the hundreds of randomized trials on drugs and therapy, only a limited number of these trials on drugs and therapy have focused on primary care patients. The results found for treatments in specialized mental health care may, however, not be valid in depressed primary care patients, also because depression is assumed to be less severe in primary care patients [9].

Although both antidepressant medication and psychotherapies have small, but positive effects on depression, with no clinically relevant differences at the short-term, many general practitioners (GPs) tend to prescribe antidepressant medications to depressed patients [10]. The majority of patients, however, prefer psychological treatments [11, 12, 13•]. In a systematic review of 34 studies among different settings, it was found that on average 75% of patients with mental disorders preferred psychotherapy over drug treatment, especially in younger patients [12], although there are indications that this may be related to severity of depression with more severe patients more often preferring drugs [14].

In this paper, we will give an overview of the available psychological treatments that have been tested in randomized trials in primary care patients. We will also examine the relative effects of therapies, antidepressants, and their combination. We will summarize what is known about how these therapies can be delivered (individually, in groups, by telephone, through the internet) and who can deliver them. Then we will focus on new developments in the field, including the use of therapies that are delivered through new technologies, the increasing number of trials on psychotherapy in primary settings in low and middle income countries (LMICs), the recent evidence that simple forms of therapy are as effective as more complicated forms, that treating subthreshold depression can have important preventive effects, and that there is increasing knowledge about the treatment of specific target groups, such as older adults, women with postpartum depression, comorbid substance use, and comorbid general medical disorders.

## Psychotherapy for Depression

In the past decades, several different types of psychotherapy for depression have been developed and tested in primary care. Psychotherapy can be defined as “the informed and intentional application of clinical methods and interpersonal stances derived from established psychological principles for the purpose of assisting people to modify their behaviors, cognitions, emotions, and/or other personal characteristics in directions that the participants deem desirable” [15].

The types of therapy that have been examined best in primary care settings are cognitive behavior therapy (CBT), behavioral activation therapy (BAT), interpersonal psychotherapy (IPT), problem-solving therapy, and non-directive counseling. In Table 1, definitions of each of these therapies are given, as well as some major sources of evidence for their effectiveness. There are many other types of therapy, which are sometimes used in mental health care but have not extensively been tested in primary care settings. Examples include brief psychodynamic therapies, life review therapy, and mindfulness-based CBT. In Table 2, the main meta-analyses of trials on psychotherapies in primary care patients have been summarized.

**Table 1:** The most important types of psychological interventions in primary care^a^

Types of therapy	Description	Evidence
Cognitive behavior therapy (CBT)	− The best examined type of psychotherapy that is currently available, also in primary care. − Although it is the best studied type of therapy, there is no evidence that it is more effective than other therapies. − The therapist focuses on the impact a patient’s present dysfunctional thoughts have on current behavior and future functioning. − CBT is aimed at evaluating, challenging, and modifying a patient’s dysfunctional beliefs (cognitive restructuring). − Therapists exert an active influence over therapeutic interactions and topics of discussion, use a psycho-educational approach, and teach patients new ways of coping with stressful situations.	− Several meta-analyses of several dozens of trials have shown that CBT is effective in primary care [16–18]. − Meta-analyses of trials across setting have included more than 200 comparisons between CBT and control groups, overall indicating comparable effects as in primary care [19].
Behavioral activation therapy (BAT)	− BAT is often combined with CBT but can also be offered as a separate treatment [19]. − The patient registers pleasant routine and essential activities. − The patient is stimulated to increase positive interactions with his or her environment. − The delivery of BAT is less complicated than CBT. − Social skills training can also be a part of the intervention.	− No meta-analysis of trials of BAT in primary care has been conducted. − Meta-analyses of BAT across settings have included several dozens of trials, resulting in comparable effects as other therapies [19, 20]. − A large non-inferiority trial found that BAT delivered by nurses is non-inferior to CBT delivered by therapists [21••].
Interpersonal psychotherapy (IPT)	− A highly structured manual-based psychotherapy that addresses interpersonal issues in depression to the exclusion of all other foci of clinical attention [20, 21••, 22]. − In secondary care, usually the full version of 16 sessions is used, but there is also a brief version interpersonal counseling (IPC) that was developed for primary care [23]. − IPT has no specific theoretical origin although its theoretical basis can be seen as coming from the work of Sullivan, Meyer, and Bowlby. − The current form of the treatment was developed by the late Gerald Klerman and Myrna Weissman in the 1980s [22].	− No meta-analysis of trials of IPT in primary care has been conducted. − Meta-analyses of IPT across settings have included several dozens of trials, resulting in comparable effects as other therapies [19, 23].
Problem-solving therapy (PST)	− In PST, patients learn how to systematically solve their problems in a number of steps. − First, the problems are defined, then as many solutions as possible are generated, the best one is chosen, a plan is made to actually do it, the plan is executed and finally evaluated whether the problem is solved. If not, the patient should go back to the first step. − PST was originally developed as a 12-session intervention which was aimed at problem-solving and also on changing attitudes and beliefs that inhibit effective problem-solving [24, 25]. − However, in the 1990s, a brief 6-session version of PST was developed specifically for primary care [26, 27].	− A meta-analysis of 11 trials of PST in primary care found comparable effects as other therapies [25]. − A meta-analysis across settings has included a few dozen of trials [19].
Non-directive counseling	− Counseling is an unstructured therapy without specific psychological techniques other than those common to all approaches, such as helping people to ventilate their experiences and emotions and offering empathy [19, 28]. − It is not aimed at solutions or acquiring new skills. − The assumption is that relief from personal problems may be achieved through discussion with others. − Often described in the literature as either counseling or (non-directive) supportive therapy. − Counseling is examined in primary care in several studies.	− No meta-analysis of trials of counseling in primary care has been conducted. − Meta-analyses of counseling across settings have included several dozens of trials, resulting in effects that may be somewhat smaller than other therapies, but this is not clear [19, 28].
Other types of therapy	There are many other types of therapy that have been tested in other settings, but not extensively in primary care: − Psychodynamic therapies are based on the psycho-analytic framework and try to help patients through resolve depression through enhancing the patient’s understanding, awareness, and insight about repetitive conflicts [29]. − Life review therapy is mainly used in older adults and is aimed at resolving conflicts from the past and make up the balance of one’s life [30, 31]. − Third wave therapies are a heterogeneous group of treatments that introduce new techniques to CBT. They have in common that they abandon or only cautiously use content-oriented cognitive interventions and the use of skills deficit models to delineate the core maintaining mechanisms of the addressed disorders [32]. − Mindfulness-based CBT, in which CBT is combined with mindfulness and meditation, is an important example. Significant effects have been obtained with this therapy in patients with diagnostic levels of depression [33], being a treatment of choice for depression recurrence recommended by NICE guidelines [34].	− For the effects of 15 different therapies, see the recent meta-analysis of Cuijpers et al. 2019 [19].

^a^The definitions given in this table are based on a broader meta-analysis of 15 evidence-based therapies for adult depression [20]

**Table 2:** Selected characteristics of meta-analyses on psychological interventions in primary care (2014–2019)^a^

	Patients	Intervention	Format	Comparison	N st	SMD	95% CI	*I*^2^	95% CI^b^
Linde, 2015 [16]	Depression	CBT	Face-to-face	TAU or placebo	7	0.30	0.13~0.48	0	0~71
Santoft, 2019 [17••]	Depression	CBT	Any	Control	34	0.22	0.15~0.30	40	18~59
Stephens, 2016 [35••]	Postpartum depr	Any	Face-to-face	TAU or WL	10	0.38	0.27~0.49	60	27~78
Twomey, 2015 [18]	Depr and anx	CBT	Any	No treatment	7	0.59	0.32~0.85	61	Nr
		CBT	Any	TAU	14	0.48	0.27~0.69	77	Nr
		CBT + TAU	Any	TAU	9	0.37	0.25~0.50	30	Nr
Wells, 2018 [36]	Depression	C-CBT	Guided + ung.	TAU or WL	8	0.26	0.07~0.45	85	73~92
Zhang 2019 [37••]	Depression	CBT, PST, MI, SFT	Any	Any comparator	65	0.42	0.29~0.56	Nr	Nr
Zhang, 2018 [38•]	Depression	PST	Face to face	Any comparator	11	0.67	0.47~0.88	Nr	Nr

*Anx*, anxiety; *C-CBT*, computerized cognitive behavior therapy; *CBT*, cognitive behavior therapy; *CI*, confidence interval; *Depr*, depression; *MI*, motivational interviewing; *N st*, number of studies; *Nr*, not reported; *PST*, problem-solving therapy; *SFT*, solution-focused therapy; *SMD*, standardized mean difference (Cohen’s *d* or Hedges’ *g*); *TAU*, treatment-as-usual; *Ung*, unguided; *WL*, waiting list

^a^Only conventional meta-analyses with at least 5 studies in a comparison and reporting a standardized mean difference are included in the table

^b^If the 95% CI of *I*^2^ was not reported, we calculated it based on the value of *Q* and *df* with the heterogi module in STATA

There is a considerable body of evidence that psychotherapies are effective in the treatment of depression. In randomized trials, therapies are usually compared with care-as-usual, waiting list, pill placebo, or other control conditions. Compared with these control conditions, the effects are moderate to large (Cohen’s *d* ranging from 0.6 to 0.9) [19]. However, the quality of these trials is in many cases suboptimal and publication bias is a major problem [39]. After adjustment for these problems, the effect sizes (Cohen’s *d*) are between 0.3 and 0.4, which corresponds with a numbers-needed-to-be-treated (NNT) between 8 and 11 [40].

There is no evidence that the effects of different types of therapy significantly differ from each other. Trials directly comparing different types of therapy [41], as well as network meta-analyses [6], suggest that all major types of therapy have comparable effects. There is one meta-analysis examining differential effects of psychotherapies conducted in primary care [42], which supports this finding of no major differences between therapies, although the number of studies was limited and some difference was found between IPT and internet-based CBT, but that was based on a small number of studies and mostly indirect evidence.

Psychotherapies in primary care can be delivered by GPs themselves, by trained nurses, by social workers, or clinical psychologists. There is hardly any research examining whether who delivers the treatment is related to the effects that are found for therapies.

## Drugs or Therapy?

Apart from psychotherapies, drugs have also been found to be effective in the treatment of depression in large-scale meta-analyses of randomized trials across settings [5••]. One meta-analysis of 17 trials of antidepressants in primary care patients found a modest, but significant effect of drugs on response compared with placebo [43••]. However, most of these trials were in patients with moderate to severe depression, while the majority of patients are in the mild to moderate range. Therefore, these findings have to be considered with caution. In addition, a large recent trial in primary care patients with moderate depression could not confirm that sertraline was more effective than placebo [44].

At the short term, the effects of psychotherapies across all settings are comparable with those of antidepressants [45••, 46, 47]. Direct comparisons between antidepressants and therapies result in small, non-significant differences [46–48], also when blinding is taken into account (when a placebo condition is included) [48], as well as sponsorship bias [49]. However, at the longer term (up to 1 year), there are indications that psychotherapy is more effective than medication [50], especially when during follow-up, the patients stop taking the medication [51]. When the patient continues to take medication during follow-up, the effects are comparable with those of CBT without continuation treatment during follow-up.

Combined treatment is more effective than either psychotherapy or pharmacotherapy alone [47], but this has been examined mostly in moderate to severe depression and it is not clear whether this is also true for milder forms of depression. Acceptability has been found to be better for psychotherapy and combined treatment compared with antidepressants alone. Most of the meta-analyses in this field have examined these issues across settings, and it has not been established whether these findings are also true in primary care.

## Technologically Delivered Therapies

One interesting development in the past years is the remote delivery of psychotherapies through the internet and mobile apps. It is becoming increasingly clear that the effects of these interventions are comparable with those of face-to-face therapies. In one recent meta-analysis of trials in which a face-to-face therapy was compared with the same intervention that was offered as an internet-based treatment, no significant differences between these formats were found [52]. However, in this meta-analysis, interventions for a broad range of mental disorders were pooled together. In another recent meta-analysis, aimed specifically at depression, the treatment format was examined in more detail [53•]. This was a network meta-analysis of 155 studies on CBT for depression in which different kinds of treatment formats were compared with each other and with different control conditions. No significant differences were found between individual, group, telephone-based, or guided self-help format [53•]. In guided self-help, the patient independently works through a standardized protocol, with support from a professional therapist. The protocol can be in book format or available on the internet, and the support from the therapist can be delivered by telephone, by email, or by chat [54]. This meta-analysis found no difference in effects of these different formats (including internet-based guided self-help). It was found, however, that although the effects of guided self-help were comparable with that of other treatment formats, the acceptability was significantly lower. Acceptability was defined as drop-out from the study for any reason. When applying guided self-help in patients, clinicians should be cautious for early drop-out in patients.

Unguided internet-based self-help is also increasingly examined in randomized trials. These are also CBT-based interventions in which the protocol is delivered over the internet, but in which no professional support is given and the patient has to go through the intervention independently. In one large “individual patient data” meta-analysis, with the primary data of 3876 patients from 13 trials on unguided internet-based self-help, it was found that the effects of these unguided interventions are smaller than those of guided self-help, but still significant compared with care-as-usual and waiting list control groups [55]. In the network meta-analysis comparing different treatment formats of CBT, self-help without any professional support was more effective than waitlist controls, but not more effective than care-as-usual. Self-help without professional support may therefore be useful as alternative for watchful waiting in mild depression, and may be effective in some patients, but the effects are not as large as those of internet-based guided self-help or face-to-face therapies [53•,^56^].

Although most of this research has been done across different settings, there are also a growing number of randomized trials in primary care. One recent meta-analysis of 8 randomized trials on computerized CBT in primary care confirmed the general findings described above that guided self-help is effective with small to moderate effects (*g* = 0.37), while unguided has little effect compared with control conditions (*g* = 0.04) [57]. This suggests that the findings across different treatment settings are also valid in primary care settings. General practitioners may, however, be less inclined to recommend low intensity interventions for their patients [58].

## LMICs

Another interesting development is that there is an increasing number of studies in primary settings in low and middle income countries (LMICS). Most research on psychotherapies in general, including therapies in primary care settings, has been conducted in high-income Western countries across North America, Europe, and Australia. Depression, however, is not only an important public health problem in these high-income countries. Although 12-month prevalence rates differ considerably across countries [59], these rates are overall very comparable in high income countries (5.5%) and LMICs (5.9%) countries [60, 61]. In China, for example, it is estimated that 54 million people suffer from depression, which is more than the total population of Spain. Depression is therefore a worldwide public health problem, with very low rates of access for people living in LMICs. Although the number of depressed people seeking help in high-income countries is also low (about 1 in 5), only one in 27 LMICs gets adequate treatment [62, 63].

Because the financial and human resources to deliver treatments for mental health problems in LMICs are typically not available, most projects on psychotherapy use a task shifting approach. Task shifting can be defined as “the rational redistribution of tasks among health workforce teams” [64], and it means that psychotherapies are delivered by trained non-specialist, lay health counselors, such as nurses or trained lay persons. Usually therapies are also very brief, often with only 4 to 6 sessions. In recent years, several randomized trials in primary care settings have been conducted using the approach, examining behavioral activation treatment in India [65–67] and Pakistan [68], problem-solving therapy through the Friendship Bench in Zimbabwe [69••], CBT in Pakistan [70], and Problem Management Plus (which combines CBT, BA, PST, anxiety management, and social support) in Pakistan [71, 72] and Kenya [73]. These efforts build on earlier work in which task shifting approaches were used in the treatment of common mental disorders [74, 75]. It should be noted, however, that not all trials in this field have found better results when compared with usual care [76, 77].

Although no meta-analysis has yet integrated the results of trials using task shifting approaches for depression in primary care settings in LMICs, the individual studies described above find positive results and suggest that this is an effective and efficient strategy to implement psychotherapies in LMICs.

## Behavioral Activation

The question whether all therapies for depression have comparable effects has been examined for several decades, and several meta-analyses have shown that the effects of different manualized therapies are indeed comparable [6, 41]. This raises the question whether therapies can be simplified without reduction of the effects. Both internet-based interventions and task shifting interventions, in which lay health counselors deliver therapies, can be seen as examples of this development, because they use considerably less resources, but have comparable effects as conventional individual therapies.

One other development in this area is research on behavioral activation treatment in primary care. CBT is the dominant type of treatment that has been recommended in most guidelines as first line treatment of depression [34, 78, 79]. However, it is also rather complicated and requires patients to be able to understand and observe their own thinking and change this. Behavioral activation is much more simple and straightforward. It only requires patients to know which activities they find pleasurable and in theoretical terms result in “an increase of positive interactions between a person and his or her environment” [18, 80]. These pleasant activities should then be integrated more into their daily life. Behavioral activation is included in most manuals for CBT, but as we saw earlier, it can also be delivered as a separate treatment without the cognitive restructuring.

The question whether CBT and behavioral activation have comparable effects goes back to the 1970s [81], and has been examined in several trials over the years [82, 83]. However, all of these trials were heavily underpowered with far too few participants to show small differential effects of either of the two [84]. In 2016, however, a large, sufficiently powered non-inferiority trial was published in which behavioral activation delivered by junior mental health workers was compared with CBT from licensed psychological therapists for depressed primary care patients [21••]. Behavioral activation treatment was found to be non-inferior to CBT. This means that it is no less effective than CBT in primary care, but is much simpler and easier for patients, and can be effectively delivered by therapists that have less training than the CBT therapists.

## Prevention

Depressive disorders that are treated in primary care are usually less severe than disorders treated in specialized mental health care. Many of these disorders would not meet the criteria for a major depressive disorder but can better be understood as subthreshold depression or minor depression. These patients have clinically relevant depressive symptoms. It has become increasingly clear that this group can benefit from preventive psychological interventions aimed at reduction of symptoms and at preventing the onset of major depression.

A recent large randomized trial in patients recruited from the community showed that a preventive internet-based intervention consisting of the combination of behavioral activation and problem-solving can have significant effects in patients with subthreshold depression [85•]. After the intervention, the level of depressive symptoms was significantly lower in the treatment group compared with the enhanced care-as-usual control group (Cohen’s *d* = 0.69). More importantly, however, at 1-year follow-up, the number of patients who developed a major depressive disorder was significantly lower in the treatment group (27%) compared with the control group (41%), with a hazard ratio of 0.59 (95% CI 0.42~0.82; *p* = 0.002) and a number-needed-to-be-treated of 5.9.

This finding is in line with previous studies showing that brief psychological interventions in people with subthreshold depression can prevent the onset of major depression at follow-up. A meta-analysis of 17 randomized trials in subthreshold depression found that the incidence rate was significantly reduced in those who received a preventive intervention compared with those who did not [86]. The incidence rate ratio was 0.74 (95% CI 0.61~0.90), which means that the incidence of depressive disorders was 26% lower in the prevention group compared with the control group. Several of these studies were conducted in primary care [87–89].

This suggests that general practitioners can play an important role in the prevention of major depression by referring patients with subthreshold depression to brief psychological preventive interventions or offering such interventions themselves. Another group of studies has focused on psychosocial interventions focused on perinatal depression, suggesting that preventive interventions may also be effective in this target group [90•].

## Specific Target Groups

Most studies examining psychotherapies in primary care patients are aimed at patients in general. However, there are also considerable numbers of studies examining psychotherapies for depression in specific target groups, such as older adults, patients with comorbid general medical disorders, such as diabetes, heart disease, or cancer, women with perinatal depression, and patients with comorbid substance use. In a large meta-analysis of 256 trials with 332 comparisons between a therapy and a control group, it was examined whether there are differences between studies in such specific target groups [91]. There was some risk of bias in more than 75% of the studies, heterogeneity was considerable in most comparisons, and the risk for an overestimate of the effect size because of publication bias was high.

Although only thirty of these studies were conducted in primary care, the studies in older adults and patients with general medical disorders did not significantly differ from studies in adults in general. For other target groups, the number of studies with low risk of bias was too small to draw any definite conclusion, although a recent meta-analysis of 10 trials in primary care on therapies in women with perinatal depression found that psychotherapy was significantly more effective than care-as-usual and waiting list control groups (Cohen’s *d* = − 0.38; 95% CI − 0.49 to − 0.27).

The effects of treatments for comorbid depression and substance use disorders are probably more modest. A meta-analysis of 20 studies in these patients found a small but significant effect of therapy on depression, but no significant effect on substance use [92]. None of these studies were conducted in primary care because these patients are usually treated in specialized mental health or substance use disorder settings.

Therapies in depressed children and adolescents have also been examined in a considerable number of studies [93], but these studies are typically conducted among children recruited from schools or who are treated in specialized mental health care. The effect sizes found for therapies in children and adolescents compared with control conditions are smaller than those in adults, especially those in children below the age of 12, although the number of studies is small and the quality of many of these studies is suboptimal [94]. It is important, however, that both the effects in children (Cohen’s *d* = 0.35) and those in adolescents (*d* = 0.55) are still significant.

## Conclusions

In this review, we saw that psychotherapies are effective in the treatment of depression in primary care patients. The effects are comparable with those of antidepressant medication in the short term and probably more effective in the longer term. Combined treatment is more effective than either psychotherapy or pharmacotherapy alone. The majority of patients prefer psychotherapy over pharmacotherapy and it is also more acceptable. In recent years, it has become clear that therapies can effectively be delivered in different formats, including internet-based formats. It can also be delivered through trained lay health counselors, as has been shown in a growing number of studies in LMICs. The type of therapy can also be simplified, as recent research comparing cognitive behavior therapy and behavioral activation has shown. Another interesting recent development is the use of psychological treatment as prevention of major depression in people with subthreshold depression. Finally, a considerable number of studies have shown that psychotherapies can effectively be used as treatment in older adults, patients with general medical disorders, and in perinatal depression. The effects are probably smaller in those with comorbid substance use problems, chronic depression, and in children and adolescents.

Although psychotherapies are effective in the treatment of depression, we have to remember that the effects are still modest as are those of antidepressant medication. The majority of patients improve during treatment, but a considerable number of these would also have improved without treatment. Improvement rates without treatment have been estimated to be about a quarter after 3 months and 50% after 1 year [95]. And on the other side of the spectrum, there is a considerable minority of about 30% of patients who do not respond to any treatment [96, 97]. Furthermore, the quality of much of the research on psychotherapies is suboptimal. So there is still a lot of room for improvement, both in terms of quality of research and in terms of treatments outcomes.

Despite these limitations, however, psychotherapy is effective and can be considered to be one of the main treatment options for depression in primary care.
